# A Classification Tree Model with Optical Coherence Tomography Angiography Variables to Screen Early-Stage Diabetic Retinopathy in Diabetic Patients

**DOI:** 10.1155/2022/9681034

**Published:** 2022-02-15

**Authors:** Hongyan Yao, Shanjun Wu, Zongyi Zhan, Zijing Li

**Affiliations:** ^1^Ningbo Eye Hospital, Ningbo University, Ningbo 315000, China; ^2^Department of Ophthalmology, Sun Yat-Sen Memorial Hospital, Sun Yat-sen University, Guangzhou 510020, China

## Abstract

**Aim:**

To establish a classification tree model in DR screening and to compare the DR screening accuracy between the classification tree model and the logistic regression model in type 2 diabetes mellitus (T2DM) patients based on OCTA variables.

**Methods:**

Two hundred forty-one eyes of 241 T2DM patients were included and divided into two groups: the development cohort and the validation cohort. Optical coherence tomography angiography (OCTA) images were acquired in these patients. The data of foveal avascular zone area, superficial capillary plexus (SCP) density, and deep capillary plexus (DCP) density were exported after automatically analyzing the macular 6 × 6 mm OCTA images, while the data of radial peripapillary capillary plexus (RPCP) density was exported after automatically analyzing the optic nerve head 4.5 × 4.5 mm OCTA images. These OCTA variables were adopted to establish and validate the logistic regression model and the classification tree model. The area under the curve (AUC), sensitivity, specificity, and statistical power for receiver operating characteristic curves of two models were calculated.

**Results:**

In the logistic regression model, best-corrected visual acuity (BCVA) (LogMAR) and SCP density were entered (BVCA : OR= 60.30, 95% CI= [2.40, 1513.82], *p* = 0.013; SCP density: OR= 0.86, 95% CI= [0.78, 0.96], *p* = 0.006). The AUC, sensitivity, and specificity for detecting early-stage DR (mild to moderate NPDR) in the development cohort were 0.75 (95% CI: [0.66, 0.85]), 63%, and 83%, respectively. The AUC, sensitivity, and specificity in the validation cohort were 0.75 (95% CI: [0.66, 0.84]), 79%, and 72%, respectively. In the classification tree model, BVCA (LogMAR), DM duration, SCP density, and DCP density were entered. The AUC, sensitivity, and specificity for detecting early-stage DR were 0.72 (95% CI: [0.60, 0.84]), 66%, and 76%, respectively. The AUC, sensitivity, and specificity in the validation cohort were 0.74 (95% CI: [0.65, 0.83]), 74%, and 72%, respectively. The statistical power of the development and validation cohorts in two models was all more than 99%.

**Conclusions:**

Compared to the logistic regression model, the classification tree model has similar accuracy in predicting early-stage DR. The classification tree model with OCTA variables may be a simple tool for clinical practitioners to identify early-stage DR in T2DM patients. Moreover, SCP density is significantly reduced in mild-to-moderate NPDR eyes and might be a biomarker in early-stage DR detection. Further improvement and validation of the DR diagnostic model are awaiting to be performed.

## 1. Introduction

Diabetic retinopathy (DR) is a serious ocular complication of diabetes mellitus (DM) that may cause irreversible blindness among the working population worldwide [1,2]. Nearly 200 million people around the world are expected to be affected by 2030 [3]. Diabetic macular edema (DME), retinal neovascularization, and tractional retinal detachment are common and severe complications of DR. Antivascular endothelial growth factor (anti-VEGF) and dexamethasone implants treatment are useful treatments for DME [4,5]. Besides, anti-VEGF has proved to be effective in retinal neovascularization [1]. As for tractional retinal detachment, pars plana vitrectomy should be adopted. However, the prognosis of these severe complications of DR is relatively poor. Early diagnosis and treatment of DR may contribute to a better visual prognosis; thus, DR screening in DM patients is crucial and necessary [6]. Manual fundus examination and fundus photography are traditional and useful methods to detect DR, but they are not quantitative and can only be determined by ophthalmologists [7]. Although many artificial intelligence (AI) products have been developed based on fundus photography, their practical uses are limited because of their relatively narrow application, complicated manipulations, and expense [8,9]. Recently, optical coherence tomography angiography (OCTA), a noninvasive cross-sectional imaging device, has been introduced in retinopathy diagnosis. The quantitative data of retinal microvasculature in different layers are obtained through one shot only without pupil dilation and are automatically analyzed and output [10]. OCTA provides an opportunity to develop prediction models to detect DR because of its objective and quantitative nature. In our previous studies and other researchers' studies [4,11–13], the vessel densities decreased more significantly as the severity degree became higher, which meant the vessel density alterations between nondiabetic retinopathy (NDR) and early-stage DR (mild and moderate non-proliferative diabetic retinopathy (NPDR)) are less obvious than those between NDR and severe NPDR or proliferative diabetic retinopathy (PDR). Therefore, it may be easier for a model to recognize the severity when referred to NDR and PDR eyes, while it may be more difficult for a model to recognize the severity when referred to NDR and early-stage DR eyes. The establishment of the latter model is crucial for the early diagnosis of DR. Clinical practitioners will be able to participate in disease early intervention and management.

Besides, most studies established prediction models using regression analyses. When assessing external data, regression analyses usually involve data input and machine calculation, which is not convenient for face-to-face clinical decisions. Recently, the classification tree model has been widely applied in clinical practice, and it helps to significantly improve the quality and efficiency of clinical decisions [14,15]. A classification tree is a nonlinear discrimination method that can split a sample into small subgroups using independent variables. This procedure chooses the most crucial variables according to specific classification rules. Clinical practitioners move through the tree by answering the questions at the branches according to patients' data and obtaining risk grades until a terminal node is present [16].

Therefore, the purpose of this study was to establish a classification tree model in DR screening and to compare the DR screening accuracy of two models, the classification tree model and the logistic regression model, in type 2 diabetes mellitus (T2DM) patients based on OCTA variables.

## 2. Methods

### 2.1. Subjects

**Figure 1:**
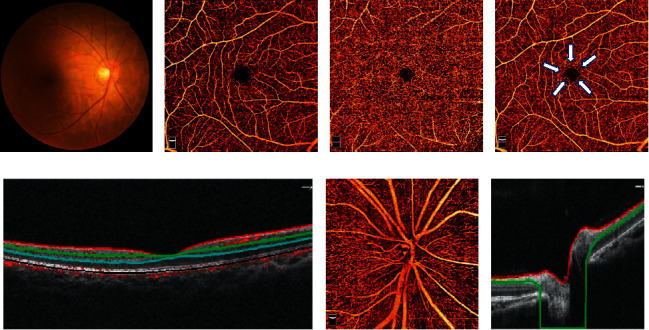
Optical coherence tomography angiography (OCTA) assessments. (a) Color fundus photographs, (b) macular 6 × 6 mm OCTA image in the superficial vascular plexus (inner plexiform layer (ILM) - inner plexiform layer (IPL): area between the red and green lines in (E)), (c) macular 6 × 6 mm OCTA image in the deep vascular plexus (IPL - outer plexiform layer (OPL): area between the green and blue lines in (e)), (d) foveal avascular zone: white arrowhead, (e) a horizontal OCT B-scan of the retina, (f) optic nerve head (ONH) 4.5 × 4.5 mm OCTA image: area between the red and green lines in (g), and (g) a horizontal OCT B-scan of the ONH.

This cross-sectional study was approved by the ethics committee of Sun Yat-sen Memorial Hospital, Sun Yat-sen University (SYSEC-KY-KS-2021-263) and was conducted according to the tenets of the Declaration of Helsinki. The medical records of 241 T2DM patients from the endocrinology and ophthalmology departments from January 2021 to June 2021 were selected and reviewed. General characteristics included age, sex, hemoglobin Alc (HbAlc) level, DM duration, and the presence of hypertension. All the patients had complete ocular examinations, including best-corrected visual acuity (BCVA), intraocular pressure (IOP) (noncontact tonometry, TX-20, Canon Inc., Tokyo, Japan), axial length (AL) (IOLMaster, Carl Zeiss Meditec, Inc., Dublin, USA), Early Treatment Diabetic Retinopathy Study 7 standard color fundus photographs (7F-ETDRS) (Canon, Inc., Tokyo, Japan), OCTA (Optovue, Inc., Fremont, CA, USA), and FFA (when necessary) (Microclear, Inc., Suzhou, China). OCTA images were acquired using AngioVue 2.0, and images with a scan quality of less than 6 were excluded. A 6 × 6 mm area centered on the fovea and a 4.5 × 4.5 mm area centered on the optic disc were captured (Figure 1). The data of FAZ area, SCP density, and DCP density were exported after automatically analyzing the macular 6 × 6 mm OCTA images, while the data of RPCP density was also exported after automatically analyzing the ONH 4.5 × 4.5 mm OCTA images.

The inclusion criteria were as follows: (1) a diagnosis of T2DM with NDR or mild to moderate NPDR; (2) age not less than 40 years; and (3) complete demographic data and ophthalmic examinations. The exclusion criteria were as follows: (1) AL longer than 26 mm; (2) intraocular pressure higher than 21 mmHg; (3) diabetic edema: central foveal thickness <300 *μ*m; (4) optic neuropathy, glaucoma, uveitis, and other retinal diseases; (5) refractive media opacity affecting imaging; and (6) a history of intraocular surgery. The DR stage was graded by two senior ophthalmologists based on the 2017 American Diabetes Association (ADA) criteria [17]. A random eye was chosen when the severity was similar, and the more serious eye was chosen when the severity differed in bilateral eyes. Two hundred forty-one eyes of these patients were included and divided into NDR and NPDR.

### 2.2. Statistical Analysis and Model Establishment

Two different methods were applied to establish and evaluate the models.

In the first model, the whole cohort was randomly divided into two groups. In cohort 1 (121 eyes), the variables between the two groups were analyzed using commercially available statistical software SPSS 24.0 (SPSS Inc., Chicago, USA). Student's *t*-tests were adopted in numerical variables, while chi-squared tests were performed in categorical variables. Statistical significance was set at *p* < 0.05. Afterward, a collinearity test was performed among the variables with significant differences. Statistically significant variables without collinearity were included in a binary logistic regression model. A backward method was adopted. Cohort 2 (120 eyes) was used to validate the model.

In another model, a classification tree method was adopted. In our study, variables with significant differences in cohort 1 were chosen, and cohort 1 was included in this classification tree model. The “rpart” package of *R* software (http://www.r-project.org) was used to establish this model. Cohort 1 was randomly divided into training sets (81 eyes) and test sets (40 eyes) several times, and a proper model was chosen based on clinical and statistical standards. The classification and regression tree algorithm was adopted, and the Gini indexes were clues to arrange the order of specific variables and to set cutoff values of each node. Cohort 2 was also used to validate the model.

**Figure 2:**
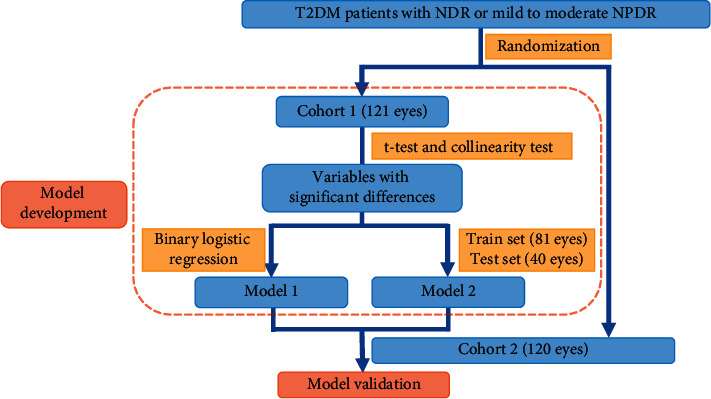
Flowchart of the development and validation in two models. T2DM, type 2 diabetes mellitus; NDR, nondiabetic retinopathy; NPDR, nonproliferative diabetic retinopathy; Model 1, binary logistic regression model; Model 2, classification tree model.

Afterwards, receiver operating characteristic (ROC) curves for the development and validation of these two models were generated, and the AUC, sensitivity, and specificity of the ROC curve were presented. Power analyses were performed in these ROC curves using the power and sample size software PASS 15.0 (NCSS, LLC. Kaysville, Utah, USA). The flowchart of development and validation in two models is shown in Figure 2.

## 3. Results

**Figure 3:**
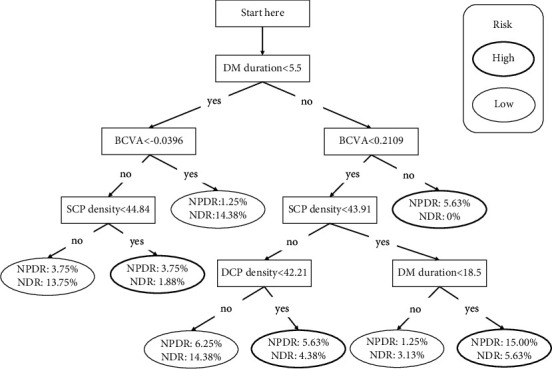
Classification tree for detecting non-proliferative diabetic retinopathy. DM, diabetes mellitus; BCVA, best-corrected visual acuity; SCP, superficial capillary plexus; DCP, deep capillary plexus; NPDR, nonproliferative diabetic retinopathy; NDR, nondiabetic retinopathy.

**Figure 4:**
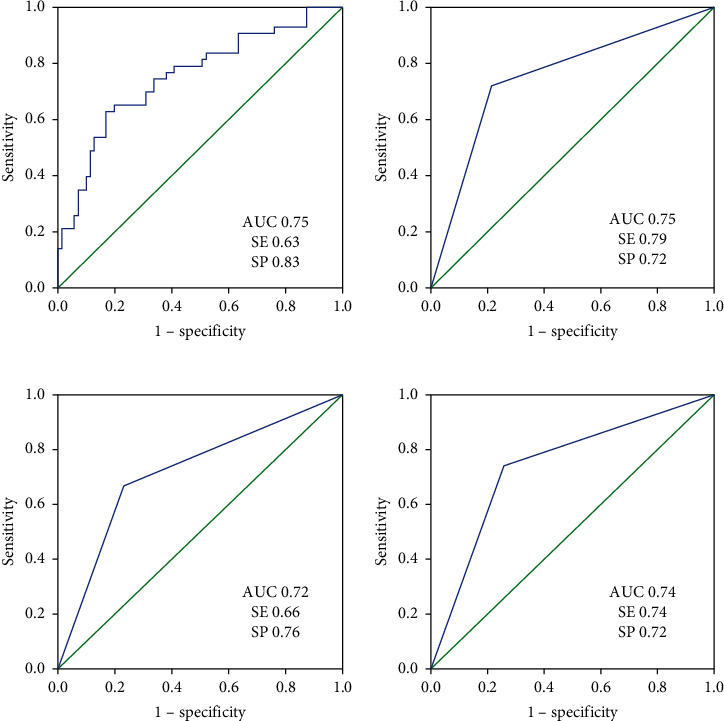
Receiver operating characteristic curve of the development cohort and validation cohort. (a) Logistic regression model development, (b) logistic regression model validation, (c) classification tree model development, and (d) classification tree model validation; AUC: area under the curve; SE: sensitivity; SP: specificity.

The general and ocular characteristics of diabetic patients in two cohorts are shown in Table 1. In the development cohort (cohort 1), DM duration and BVCA (LogMAR) were significantly lower in patients with NDR compared with those with NPDR, whereas SCP, DCP, and RPCP density were significantly higher in patients with NDR. There was no significant difference in the remaining variables. No collinearity was found among DM duration, BVCA (LogMAR), SCP, DCP, and RPCP density. Therefore, these 5 variables were included in the binary logistic regression model and the classification tree model. In the former model, BVCA (LogMAR) and SCP density were entered (BVCA : OR= 60.30, 95% CI= [2.40, 1513.82], *p* = 0.013; SCP density: OR= 0.86, 95% CI= [0.78, 0.96], *p* = 0.006). Details are shown in Table 2. The AUC, sensitivity, and specificity for detecting early-stage DR (mild to moderate NPDR) in the development cohort were 0.75 (95% CI: [0.66, 0.85]), 63%, and 83%, respectively. Besides, the AUC, sensitivity, and specificity in the validation cohort were 0.75 (95% CI: [0.66, 0.84]), 79%, and 72%, respectively. In the latter model, BVCA (LogMAR), DM duration, SCP density, and DCP density were entered, and the classification tree is shown in Figure 3. The AUC, sensitivity, and specificity for detecting early-stage DR were 0.72 (95% CI: [0.60, 0.84]), 66%, and 76%, respectively. Besides, the AUC, sensitivity, and specificity in the validation cohort were 0.74 (95% CI [0.65, 0.83]), 74%, and 72%, respectively. The ROC curves for these two models are shown in Figure 4. Detailed validation outcomes and predictors in two models are shown in Table 3.

**Figure 5:**
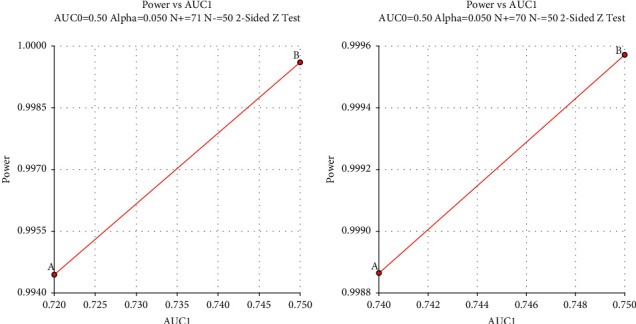
Power analysis results for the development cohort and validation cohort. (a) Development cohort; (b) validation cohort; (A) classification tree model; (B) logistic regression model.

With regard to the power analyses, in the development cohort, a sample of 71 from the NPDR and 50 from the NDR group achieves 99.44%/99.96% power to detect a difference of 0.22/0.25 between the AUC under the null hypothesis of 0.50 and an AUC under the alternative hypothesis of 0.72/0.75 using a two-sided *z*-test at a significance level of 0.05, respectively, in the binary logistic regression model and the classification tree model. Similarly, in the validation cohort, a sample of 70 from the NPDR and 50 from the NDR group achieves 99.89%/99.96% power to detect a difference of 0.24/0.25 between the AUC under the null hypothesis of 0.50 and an AUC under the alternative hypothesis of 0.74/0.75 using a two-sided *z*-test at a significance level of 0.05, respectively, in the binary logistic regression model and the classification tree model. All the data are continuous responses. The AUC is computed between false positive rates of 0.00 and 1.00. The ratio of the standard deviation of the responses in the NDR group to the standard deviation of the responses in the NPDR group is 1.00. The power analysis results are shown in Figure 5.

## 4. Discussion

In previous studies on DR screening [18–20], demographical information, disease information, and biochemical indicators were major variables in DR prediction models. In our study, DM duration was included in the classification tree model but not included in the logistic regression model because of a collinearity between DM duration and BCVA existed in the regression analysis. A regression analysis may extract the major components in prediction but may lose detailed valuable information [21]. Therefore, a classification tree model seems to be more appropriate because it can keep more clues and evidence to support a diagnosis. Moreover, in other studies on DR screening [18,19], the risk variables for the occurrence of DR were diverse. The alterations among these models or studies imply that the construction of a statistical model may be affected by a series of factors. These factors not only included the statistical problems such as collinearity among the variables, but also included the cohort characteristic alterations. Therefore, the included risk variables may be distinct in different screening models because the study cohorts have various demographical and disease characteristics. Ethnicity may have contributed to the difference. DM duration, insulin treatment, HbA1c level, and microalbuminuria were major risk contributors in a Spanish study [18], while the presence of hypertension, DM family history, and low frequency of physical activity were risk factors in a Danish study [22]. Besides, our study was a hospital-based study, and the included patients might have more serious and complicated conditions than patients in communities or regions outside hospitals. This might also be an explanation for the alterations between other studies and ours, even performed in the same country [23].

Different methods were adopted in these models, and the models acquired relatively high accuracy. Shen and his colleagues [23] proposed a model using the XGB-Stacking algorithm based on improved backward search, and the highest accuracy was 83.95%. Similarly, in Romero–Aroca's study [18], a clinical decision support system based on a fuzzy random forest was utilized and an accuracy of 87.6% was obtained. Although proper models have been established using advanced algorithms, these models solely focus on the systemic conditions of T2DM patients.

A more straightforward method to screen DR is fundus imaging. Our present study is a combination of systemic conditions and fundus assessments. Herein, a binary logistic regression model and a classification tree model were developed to detect early-stage DR, and the results showed similar AUCs and accuracies in detection. In our study, a novel quantitative machine called OCTA was adopted. It is a noninvasive, convenient, and promising machine frequently used in DR diagnosis. According to the regions and layers of the retina, the microvasculature can be divided into different capillary plexuses. A certain capillary plexus provides blood supply for a certain region and layer [24]. SCP, DCP, and RPCP have been proved to be sensitive variables in DR severity assessment [25,26]. Fundus photography is a common and traditional tool in DR screening [7]. Many studies yielded an accuracy of more than 80% in their proposed models [27,28]. Texture was adopted to model the establishment in Acharya's study [29]. An accuracy of 85.2%, a sensitivity of 98.9%, and a specificity of 89.5% were achieved. Features based on exudate area, blood vessels, texture, and entropies were used to detect DR in Mookiah's study [30]. An accuracy of 92.88%, a sensitivity of 96.27%, and a specificity of 96.08% were yielded by the probabilistic neural network classifier. Mane [27] set up a holoentropy enabled-decision tree model. The accuracy, sensitivity, and specificity were 96.45%, 96.72%, and 97.01%, respectively. The algorithm alterations may exert an influence on the accuracy in some degree. Nevertheless, because fundus photography is not inherently quantitative, it can only be assessed by ophthalmologists or AI techniques [31]. Therefore, though the accuracy of our models was less than that of other models based on fundus photography, OCTA, as a quantitative device, may still be a potentially extraordinary technique for model establishment. In recent years, researchers have made efforts to update DR screening strategies besides using fundus photography. Deng [32] integrated a quantitative device called a handheld electroretinogram with the classification tree model and found good accuracy. Moreover, handheld smartphone-based retinal cameras were applied in urban primary healthcare settings and the accuracy maintained above 80% [33,34]. Studies on DR screening using advanced quantitative devices are dramatically increasing, but most of them are awaiting further improvements. In the future, if the simplification of the imaging and analysis process for OCTA has been improved, OCTA may be increasingly acceptable for DR prediction. A combination of OCTA and the classification tree model will be of great use in DR screening. Our study was the first primary attempt to complete the combination.

SCP density was included both in the logistic regression model and the classification tree model, while DCP was only included in the classification tree model. Moreover, SCP was at a higher node than DCP was in the classification tree. Generally, SCP mainly connects with retinal arterioles while DCP mainly connects with retinal venules [24]. SCP may have a stronger self-regulatory capacity than DCP, and DCP is more vulnerable in DR. In Simonett's study [35], a reduction was found in DCP density but was absent in SCP density; however, Tian and our studies [36] confirmed significant differences in SCP. In our study, although significant differences existed in both SCP density and DCP density, SCP density seemed to be more extraordinary in prediction. One possible reason is that SCP projects flow signal artifacts onto the DCP and affects the accuracy of DCP density.

We noticed an interesting phenomenon in the classification tree: when SCP density was less than 43.91%, a DM duration of less than 18.5 years seemed to be a risk factor. This finding might suggest that a long DM duration does not necessarily accelerate DM development because DM development is also closely related to blood glucose control and other factors [18]. Moreover, the percentage of patients with a DM duration of not less than 18.5 was small, and the difference may not be obvious. Besides, in the binary logistic regression model, the 95% CI was large. As we have performed a collinearity test among the statistically significant variables and no collinearity was found among them, we may contribute the reason to the relatively small sample and scattered data in BCVA. Clinically, BCVA alterations between NDR and mild-to-moderate NPDR may be less obvious than those between NDR and PDR, therefore required lager data. However, we finally included BCVA as a predictor because BCVA may be a basic and crucial variable in present clinical practice. Moreover, we finally validated and evaluated the model, and the outcome was relatively satisfactory. However, more well-designed studies should be performed to further improve and validate the model.

There were some limitations in this study. First, the classification tree model is a general and rough prediction and cannot make very precise decisions. Second, the sample size was relatively small and hospital-based, which may have caused some bias. The absence of evidence in difference of AUC between the two models may be due to the small sample size in this study. Moreover, this was a retrospective study, and some other variables were not included. A prospective cohort with a large population needed to improve and validate the model.

## 5. Conclusions

Compared to the logistic regression model, the classification tree model has similar accuracy in predicting early-stage DR. The classification tree model with OCTA variables may be a simple tool for clinical practitioners to identify early-stage DR in T2DM patients. Moreover, SCP density is significantly reduced in mild-to-moderate NPDR eyes and might be a biomarker in early-stage DR detection. Further improvement and validation of the DR diagnostic model are awaiting to be performed.

## Figures and Tables

**Table 1:** General and ocular characteristics of diabetic patients in two cohorts.

	Cohort 1			Cohort 2		
	NDR	NPDR	*Pvalue*	NDR	NPDR	*Pvalue*
Eyes (n)	71	50	NA	70	50	NA
Age (years)	56.39 ± 11.01	57.00 ± 8.83	0.749	56.83 ± 10.62	57.61 ± 9.12	0.676
Sex (male: female)	43 : 28	31 : 19	0.873	41 : 29	26 : 24	0.475
HbAlc (%)	8.63 ± 2.17	8.83 ± 2.45	0.640	8.59 ± 2.09	9.22 ± 2.56	0.159
DM duration (years)	7.27 ± 6.77	11.46 ± 7.58	0.003^*∗*^	7.64 ± 6.92	11.50 ± 6.94	0.006^*∗*^
Presence of hypertension (n, %)	6, 8.45%	5, 10.00%	1.000	4, 5.71%	5, 10.00%	0.598
Severity (mild: moderate)	NA	20 : 30	NA	NA	24 : 26	NA
IOP (mmHg)	15.51 ± 2.52	15.38 ± 3.69	0.822	15.48 ± 2.72	15.17 ± 3.63	0.600
BVCA (LogMAR)	-0.0079 ± 0.11	0.10 ± 0.21	<0.001^*∗*^	0.0020 ± 0.10	0.072 ± 0.19	0.01^*∗*^
FAZ area (mm^2^)	0.33 ± 0.12	0.37 ± 0.24	0.153	0.37 ± 0.26	0.41 ± 0.27	0.443
SCP density (%)	46.79 ± 3.83	43.71 ± 4.72	<0.001^*∗*^	46.51 ± 4.22	43.80 ± 4.32	0.001^*∗*^
DCP density (%)	46.18 ± 6.12	43.09 ± 6.32	0.008^*∗*^	45.52 ± 4.87	42.17 ± 6.18	0.001^*∗*^
RPCP density (%)	48.02 ± 3.13	46.43 ± 3.04	0.007^*∗*^	47.49 ± 4.00	46.27 ± 3.04	0.078

NDR, nondiabetic retinopathy; NPDR, nonproliferative diabetic retinopathy; NA, not available; DM, diabetes mellitus; IOP, intraocular pressure; BCVA, best corrected visual acuity; FAZ, foveal avascular zone; SCP, superficial capillary plexus; DCP, deep capillary plexus; RPCP, radial peripapillary capillary plexus. ^*∗*^*P* <0.05.

**Table 2:** Regression coefficients in the binary logistic regression model.

	B	SE	Wald	*P* value	OR	95% CI for OR	
						Lower	Upper
BVCA (LogMAR)	4.10	1.64	6.21	0.013^*∗*^	60.30	2.40	1513.82
SCP density	−0.15	0.053	7.71	0.006^*∗*^	0.86	0.78	0.96
Constant	6.06	2.40	6.37	0.012^*∗*^	428.50		

B, regression coefficient; SE, standard error; OR, odds ratio; CI, confidence interval; BCVA, best-corrected visual acuity; SCP, superficial capillary plexus;^*∗*^*P* <0.05.

**Table 3:** Validation outcomes and predictors in two models.

	Binary logistic regression model		Classification tree model	
	Predicted NPDR (*n*)	Predicted NDR (*n*)	Predicted NPDR (*n*)	Predicted NDR (*n*)
True NPDR (*n*)	55	15	52	18
True NDR (*n*)	14	36	14	36
Predictors	BVCA (LogMAR) and SCP density		BVCA (LogMAR), DM duration, SCP density, and DCP density	

NPDR, nonproliferative diabetic retinopathy; NDR, nondiabetic retinopathy; BCVA, best-corrected visual acuity; SCP, superficial capillary plexus; DM, diabetes mellitus; DCP, deep capillary plexus.

## Data Availability

Data supporting this research article are available from the corresponding author on reasonable request.
